# Light-emitting diode street lights reduce last-ditch evasive manoeuvres by moths to bat echolocation calls

**DOI:** 10.1098/rsos.150291

**Published:** 2015-08-05

**Authors:** Andrew Wakefield, Emma L. Stone, Gareth Jones, Stephen Harris

**Affiliations:** School of Biological Sciences, Life Sciences Building, University of Bristol, 24 Tyndall Avenue, Bristol BS8 1TQ, UK

**Keywords:** artificial lighting, light-emitting diode, street lights, bats, moth predation

## Abstract

The light-emitting diode (LED) street light market is expanding globally, and it is important to understand how LED lights affect wildlife populations. We compared evasive flight responses of moths to bat echolocation calls experimentally under LED-lit and -unlit conditions. Significantly, fewer moths performed ‘powerdive’ flight manoeuvres in response to bat calls (feeding buzz sequences from *Nyctalus* spp.) under an LED street light than in the dark. LED street lights reduce the anti-predator behaviour of moths, shifting the balance in favour of their predators, aerial hawking bats.

## 1. Introduction

Globally, lighting contributes 1900 million tonnes of CO_2_ to the Earth's atmosphere annually [1]. In 2009, approximately one-third of UK street lights were due to be upgraded [2], mainly from mercury vapour (MV) and sodium lights to energy-saving technologies such as light-emitting diodes (LEDs). While this may reduce global energy consumption, these lights are being installed *en masse* without adequate research to establish their effects on human health and wildlife [2–4]. The change from sodium street lights that emit narrowband spectra to broad-spectrum ‘white’ lights is likely to alter a wide range of species interactions [5,6].

**Figure 1.**
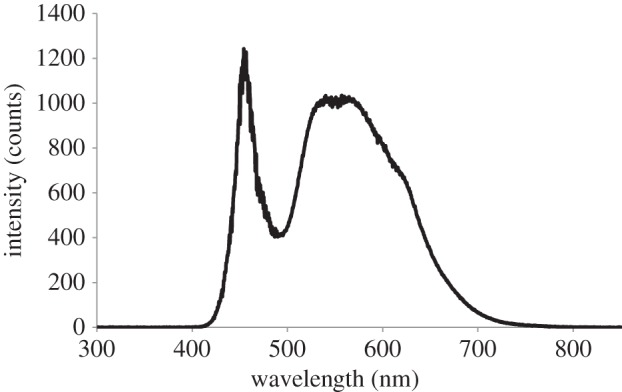
Spectral distribution of the Philips Mini Iridium LED street light.

In Britain, many moth species have suffered major population declines [7]. Light pollution is a potential driver behind these declines [8]; one-third of flying insects attracted to artificial lights die [9]. Predation of insects around artificial lights by bats is also well documented [10–13]. Although many insects have tympanic ears to detect predatory bats [14], ultraviolet (UV)-emitting, broad-spectrum MV street lights reduce a moth's likelihood of responding to bat echolocation calls [12,15] and increase the risk of predation [16]. We used field experiments to test the hypothesis that UV-absent, broad-spectrum LED street lights (figure 1) also impair the ability of moths to elicit evasive ‘powerdive’ flight behaviours in response to the echolocation calls of foraging bats.

## 2. Material and methods

### 2.1 Study sites and lighting

We conducted experiments between 29 July and 5 September 2013 at four sites around Bristol, UK that were free from artificial lighting. A commercially available street light luminaire (Mini Iridium, Philips Lighting UK) fitted with a panel of 24 LEDs was top-mounted on a 4 m high tripod (REF 49-Z, Powerdrive Drum Company Ltd., Bedfordshire, UK) using a custom-made aluminium adaptor to conform with the light's mounting specifications. The light was positioned along field-woodland boundaries, directed away from the woodland into the open grassland (electronic supplementary material, figure S1) and powered by a Honda Eu10i portable generator (Honda, Slough, UK) positioned on average 81 m away (range=68–90 m). Irradiance measurements of the light were taken in a darkened room using a cosine corrector at the end of a 400 μm diameter UV-visible fibre-optic cable connected to a spectrometer (USB2000, Ocean Optics, FL, USA) controlled by a PC running SpectraSuite (v. 6, Ocean Optics). The integration time was set to 4 ms, and the curve represents the average of 20 scans. The end of the fibre with the cosine corrector was positioned 162 cm away, directly below the LEDs (figure 1).

### 2.2 Treatments

Defensive behaviour of moths was tested across four randomized treatments, with one treatment per night (table 1). Illuminance (lux) was recorded for each moth using a T-10 illuminance meter (Konica Minolta Sensing Inc, Osaka, Japan) mounted on a custom-built, measuring pole used to record flight height (m). The horizontal distance from the pole to the street light column was measured to the nearest 50 cm. Weather data (temperature, humidity and wind speed) were recorded every 5 min using a remote weather station (Watson W-8681-SOLAR, Flightstore Pilot Supplies Limited, Yorkshire, UK). Celestial data (lunar illumination) were obtained from http://www.timeanddate.com/.

**Table 1.** Experimental treatments.

control	street light off, no bat echolocation calls played
bat	street light off, moths exposed to bat echolocation calls
LED	LED street light on, no bat echolocation calls played
LED-bat	LED street light on, moths exposed to bat echolocation calls

### 2.3 Bat echolocation recordings

**Figure 2.**
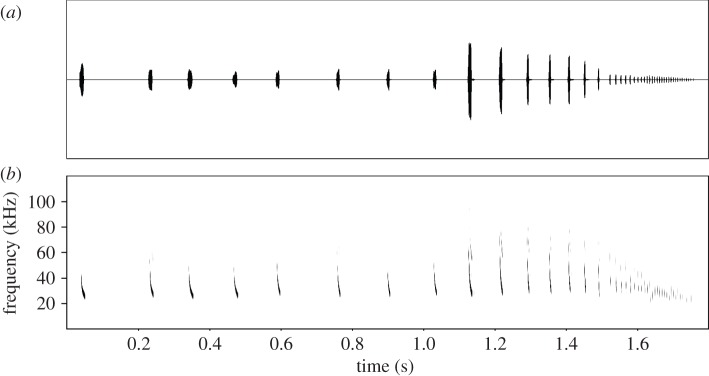
Waveform (*a*) and spectrogram (*b*) of one of the 30 pre-recorded bat echolocation call sequences (edited with SASLAB PRO (Avisoft Bioacoustics, Berlin, Germany; FFT size 1024)). Playback sequences were edited from calls where the search-phase component of the sequence recording (not shown) was identified as *Nyctalus* spp. based on existing echolocation parameters including: frequency of maximum energy (kHz), start frequency (kHz), end frequency (kHz), call duration (ms) and interpulse interval (ms) [18,19]. British *Nyctalus* spp. typically broadcast their loudest calls between 19 and 27 kHz [19], as is the case with our recordings.

Bat echolocation calls produced by *Nyctalus* *noctula* were recorded at Blagdon Lake (Somerset, UK) between 6 May and 27 June 2013 using a Pettersson D1000X ultrasound bat detector (Pettersson Elektronik AB, Uppsala, Sweden). It is possible that some calls emitted by the much rarer *Nyctalus leisleri* were recorded, as the calls of the two species are often impossible to discriminate. Both species emit similar calls with dominant frequencies that are close to the best hearing frequencies of many moths, and both feed to some extent on Lepidoptera [17]. Recordings of ‘feeding buzzes’ produced in response to small pebbles thrown into bat flight paths were made at 384 kHz and at 16-bit (see the electronic supplementary material). Call sequences were analysed in SASLab Pro (Avisoft Bioacoustics, Berlin, Germany). Sequences were trimmed to include the final few search phase calls, as well as the entirety of the approach phase and terminal buzz (figure 2).

Thirty call sequences were saved to a playlist on the D1000X detector for playback experiments using a Pettersson L400 ultrasound speaker with a frequency range of 10–110 kHz (Pettersson Elektronik AB, Uppsala, Sweden). Sequence order was randomized and sequences were not repeated on the same night. The output intensity of the speaker was calibrated prior to field experiments (see the electronic supplementary material). The mean maximum output of the speaker across sequences was 107 dB peSPL at 1 m (range=103–109 dB), equivalent to a source level of 127 dB peSPL at 10 cm, which corresponds closely with the source levels of bat echolocation calls [20–22] and is comparable in intensity to the ‘dog whistle’ used in earlier studies on moth defence behaviour [15,23].

### 2.4 Behavioural recordings

Flight behaviour of moths was recorded using a HXR NX70 professional video camera (Sony UK, Weybridge, UK) under infrared lighting (Aegis, 30° 850 nm, Bosch, Stuttgart, Germany). Moths could not be identified to species as they flew too high, fast or unpredictably for capture. However, all were relatively large and probably belonged to the families Geometridae, Noctuidae or Notodontidae. Moths in these families possess ultrasonic hearing [24] and comprised more than 90% of moths attracted to artificial lights in southwest England [6]. The video camera, infrared light, D1000X bat detector and ultrasonic speaker were mounted on custom-made housing (electronic supplementary material, figure S2) at approximately 1.4 m and aimed towards subject moths. An additional frequency division bat detector (Batbox Duet, Steyning, West Sussex, UK) was attached to the housing to ensure moths were not responding to calls from bats flying in the vicinity.

Video recordings were made 4 m from the street light column at 90° to the edge of the woodland. All moths were tested at a height of 2–4 m, within 5 m of the street light column and 4 m (±1 m) horizontally from the speaker. Moths flying less than 3 m or more than 5 m from the speaker were excluded. Call intensities likely to have been heard by moths less than 3 m were more intense than those produced by wild bats and therefore may have unrealistically influenced moth evasive responses. Moths flying more than 5 m from the speaker may not have heard the bat calls. Each playback presentation was initiated once a moth was within 5 m of the speaker and therefore in an appropriate position to hear the recordings. Ultrasonic pulses (produced by an electronic dog whistle (27 kHz, 110 dB at 1 m) and comparable in frequency and intensity to those used in our study) emitted less than 5 m away caused moths to spiral or dive towards the ground, whereas pulses emitted 5–12 m away caused moths to alter the direction of their flight path [23]. These are typical of the ‘A2’ and ‘A1’ responses of tympanate moths, respectively [25]. Our aim was to elicit A2 evasive behaviours as they are easier to distinguish from the erratic flight movements that moths typically display near street lights than A1 responses [26]. Using a mean speaker output of 127 dB (peSPL at 10 cm), the maximum call intensity at the moth's ear was estimated at approximately 93–98 dB. These values are comparable with earlier studies [20] and exceed the minimum hearing threshold of many tympanate moths [21,27].

### 2.5 Flight behavioural analysis

Moth video recordings were analysed manually by eye ‘frame-by-frame’ and scored in a similar way to [23]. Responses were classified as: (i) no change in flight; (ii) a single change or a rapid series of changes in flight course (zig-zag flight), in either case not towards the ground, i.e. presumed A1 response; (iii) a rapid dive (straight vertically) or a spiralling flight (not straight) towards the ground, i.e. presumed A2 response; and (iv) undetermined (behaviour unclear).

### 2.6 Statistical analyses

Owing to the difficulty in distinguishing sound-induced directional changes from light-induced flight erraticism, moth flight responses from category (ii) were pooled with category (i) and compared to category (iii) to test the hypothesis that moth powerdive behaviour decreases under LED lights. Pooled data were analysed using log-linear generalized linear mixed models (GLMM) with binomial errors in R (v. 2.15.1, 2012), using the package lme4 [28]. Site was included as a random effect. Treatment type was included as the only significant (*p*<0.05) fixed effect term following model simplification using backwards multiple regression (see the electronic supplementary material).

## 3. Results

**Figure 3.**
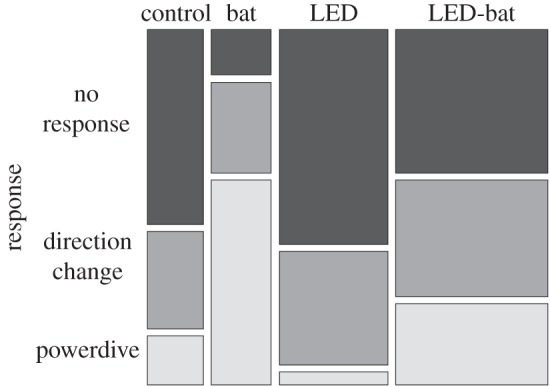
Mosaic plot illustrating the overall proportion of moth flight responses in relation to treatment type. Column widths are proportional to sample sizes.

Data from 94 moths (control *n*=14; bat *n*=15; LED *n*=27; LED-bat *n*=38) from 16 nights at four different sites were included. For each model, data from 39 moths were omitted from analyses because they were either flying more than 5 m from the street light column; flying less than 3 m from the ultrasonic speaker; flying greater than 5 m from the ultrasonic speaker; or because echolocation calls from wild bats were heard clearly on the bat detector during treatments. A further 18 recordings were excluded as these moths' responses were placed in category (iv) (behaviour unclear). The number of powerdives was significantly lower under ‘LED-bat’ treatments than ‘bat’ treatments (s.e.=0.683, *z*=−2.393, *p*=0.012) but significantly higher than under ‘LED’ treatments (s.e.=1.136, *z*=1.987, *p*=0.047). Only 24% of moths performed powerdives during ‘LED-bat’ treatments compared with 60% during ‘bat’ treatments (figure 3). The proportion of powerdives was significantly higher for ‘bat’ treatments than both bat-free treatments (‘control’, s.e.=1.198, *z*=2.405, *p*=0.016; ‘LED’, s.e.=1.198, *z*=3.249, *p*=0.001) and was not significantly different between the two bat-free treatments (s.e.=1.328, *z*=−1.168, *p*=0.243).

## 4. Discussion

Our results show that LED street lights negatively affect the probability that moths will exhibit powerdives in response to hunting bats, thereby reducing their ability to evade predation. In a previous study, approximately half as many moths reacted to ultrasound under MV lights than in the dark [15]. Similarly, we found that less than half as many moths performed powerdives in response to bat echolocation calls (60% versus 24%) under LED street lights. Thus, LED lights reduce predator-avoidance responses in moths to an extent similar to that determined at MV lights currently being phased out, despite the absence of UV emissions in LED lights.

While we were unable to identify individual species of moth tested in this study, the majority probably belonged to the families Geometridae, Noctuidae or Notodontidae, which possess ultrasonic hearing [24] and constitute the vast majority (more than 90%) of moths attracted to artificial lights in southwest England [6]. Our experimental design, comparing relative powerdive activity under LED-lit and -unlit treatments in a randomized design, controls for the possibility that a minority of moths in each treatment were not able to hear the ultrasonic playbacks.

The underlying cause of this inhibition is unclear. The small china-mark moth *Cataclysta lemnata* alters its defence strategy between day and night [29]. Reacting unnecessarily to anthropogenic and orthopteran ultrasounds during the day could be energetically disadvantageous compared to moths which ‘switch-off’ their ultrasound responses. If artificial lighting causes moths to behave as if they are flying during daylight, this could explain our results.

We need a better understanding of how different taxa respond to various artificial lights as negative impacts are likely to have cascading ecosystem effects. Globally, insects perform a vital role in providing ecosystem services such as pollination, nutrient decomposition and pest control, as well as being prey for many species. While LED street lights are detrimental to populations of tympanate moths through reduced predator avoidance, they may be more ‘insect-friendly’ than other street lighting technologies (e.g. metal halide, MV, high-pressure sodium) as they lack UV, which causes ‘flight-to-light’ behaviour and high levels of mortality [30,31].

## Supplementary Material

Title Electronic supplementary material - LED street lights reduce last-ditch evasive manoeuvres by moths to bat echolocation calls Description Electronic supplementary material providing further detail to experimental methods and results.

## Supplementary Material

Title Raw dataset - Electronic supplementary material. Description Tabulated raw data set.

## Data accessibility

Supporting data are supplied in the accompanying electronic supplementary material. R software for statistical computing and graphics can be found at http://www.r-project.org.
