# Supplementary material for: Light-emitting diode street lights reduce last-ditch evasive manoeuvres by moths to bat echolocation calls
Source: R Soc Open Sci. 2015 Aug 5;2(8):150291. doi: 10.1098/rsos.150291 (PMC4555863; doi:10.1098/rsos.150291)
Supplement: Title Electronic supplementary material - LED street lights reduce last-ditch evasive manoeuvres by moths to bat echolocation calls Description Electronic supplementary material providing further detail to experimental methods and results. [file rsos150291supp1.doc]

**Electronic Supplementary Material - LED street lights reduce last-ditch evasive manoeuvres by moths to bat echolocation calls**

**Authors**

Andrew Wakefield, Emma L. Stone, Gareth Jones and Stephen Harris

School of Biological Sciences, Life Sciences Building, University of Bristol, 24 Tyndall Avenue, Bristol, BS8 1TQ

**Supplementary Methodology**

**Sites**

Individual sites were at least 2 km apart and comprised of open grassland adjoining mixed woodland. Habitat type within each site was quantified within a 45 m radius from the lighting unit using aerial imagery and ground-level assessments. Sites were selected so that approximately 50 % of ground area was covered with mixed woodland and approximately 50 % was maintained as open grassland. Field-woodland boundaries were chosen to mimic habitats in which rural street lighting installations occur, with linear field margins representing the open space of a minor road.

**Bat Echolocation Recordings**

The detector was set to the following settings: Fs = 384.0 kHz, TE = x10, HP = OFF, where, Fs is sampling frequency, TE is the time expansion factor, and HP refers to the high-pass filter. On average call sequences were 1.75 s in duration (range 1.0 – 2.6 s). Call sequence volume was normalised to 50 % to ensure that the loudest call was the same amplitude between sequences. High-pass filters (15 kHz) were applied to call sequences to remove any non-bat noises made during recordings. Some (n = 11) of the sequences were edited using individual band-stop filters to remove unwanted, overlapping calls from *Pipistrellus* spp. Prior to field experiments the output intensity of the speaker was calibrated from the loudest call from each sequence using an acoustic calibrator (D-1411E, Dawe Instruments, London, UK), an oscilloscope (54602B 150 MHz Oscilloscope, Hewlett-Packard Limited, Bracknell, Berkshire, UK), a ¼ inch free-field microphone (Model 2520, Larson Davis Laboratories, Utah, USA) and the equation: peSPL = 114 dB + 20.log10 (Vmeas/Vref) [1], where, peSPL is the peak equivalent sound pressure level at 1 m, Vmeas is the mean signal from five oscilloscope readings recorded from each call sequence, and Vref is the reference signal produced by the acoustic calibrator.

**Treatments**

During treatments 1 and 3 (see main text, table 1) no pre-recorded bat call sequences were broadcast. Instead a functionless button was pressed on the D1000X detector to mimic the same sequence of events during all treatments. When no sound was heard on the Batbox Duet during treatments where moth flight behaviour could be synchronised during video playback analyses, the operator said “now” as the button on the detector was depressed. This was consistent across all four treatments. Previous studies investigating moth defensive behaviour have used a head-torch to locate moths during dark/unlit treatments [2,3]. We used an infrared light and video camera to detect the presence of moths to minimise the risk of influencing the moth's behaviour. While some Lepidoptera possess red visual receptors, most have UV, blue and green photo receptors [4] and so are less likely to respond to an infrared light than a white-light head-torch. For consistency, the infrared light remained on during both unlit and lit treatments.

**Statistical Analyses**

1. **Powerdive Analysis**

The following terms were non-significant (p > 0.05) during model simplification [5] and were excluded from the minimum adequate models: wind speed; humidity; temperature; the distance of moths to the lighting column; the distance of moths to the speaker; the height of moths above the ground; time elapsed between sunset and each moth response test; lunar illumination data and Julian day. Three models were compared using the change in deviance and difference in Akaike Information Criterion (AIC) values [5] (table S1). GLMM2 was a significantly better fit than GLMM1 (Δ deviance on removing treatment = 18.754, n = 94, d.f. = 3, p < 0.001; AIC for model without treatment = 101.89, vs. 89.14 with the term), and a better fit than GLMM3 which excluded any random effects.

1. **Response Analysis**

In a separate analysis, flight responses classed in categories 2 and 3 were pooled and compared with category 1 data to test the hypothesis that moths showed some form of reaction (either powerdive or flight direction change) to bat echolocation calls.

**Supplementary Results**

1. **Powerdive Analysis**

A sub-sample of 20 moth responses were classified blind by an independent scorer using the same scoring criteria (see main text). 18 responses were classified identically to the original scorer. The two discrepancies were: (i) a ‘change in flight direction’ response (category 2) reclassified as ‘no change in flight course’ (category 1), and (ii) a ‘powerdive’ (category 3) reclassified as ‘behaviour unclear’ (category 4). Statistical analyses were re-run having modified the original dataset to match with the independent scorer’s classification. Alterations included substituting discrepancy number 2 (above) as well as changing at random another behaviour originally marked as a ‘powerdive’ (category 3) to ‘behaviour unclear’ (category 4). Modifying these two responses is a fair adjustment as only one powerdive out of 10 presented to the independent scorer was misidentified and 21 moth responses were originally classified as powerdives. As the main statistical analyses compared frequency of powerdives against no powerdives (pooled category 1 and 2 data) it was not necessary to make changes following the first of the two discrepancies (category 2 to category 1). Since statistical significance of our results did not change using the altered dataset, we have confidence in our results.

The median light level for moths that performed powerdives was 0.09 lux, the median value for those moths which did not perform powerdives was 128.2 lux. Light measurements were taken at the position each moth was flying when exposed to the recorded bat calls using a T-10 illuminance meter (Konica Minolta Sensing Inc, Osaka, Japan). Field measurements were taken in lux as the spectral output of our light (see main text, figure 1) was almost exclusively in the human visual range (400 - 700 nm).

1. **Response Analysis**

Robustness of the minimum adequate model for the moth behavioural response data was tested using model comparisons. The model with site included as a random effect had a lower AIC than a generalized linear model including treatment as a fixed effect but excluding any random effects (AIC: 124.59 vs. 126.22). The model with site as a random effect and treatment as a fixed effect was a significantly better fit to the data than the model without the fixed effect (Δ deviance on removing treatment = 12.075, d.f. = 3, p = 0.007; AIC for model without treatment = 130.67, vs. 124.59 with the term). Fewer moths responded on ‘LED-Bat’ nights compared with ‘Bat’ treatments (table S2), although the difference was not statistically significant (SE = 0.8641, z = -1.886, p = 0.059). The proportion of moths responding was larger in comparison to the ‘LED’ treatment, although not statistically significant (SE = 0.5466, z = 1.764, p = 0.078). 58 % of moths performed either a powerdive or changed their flight direction during ‘LED-Bat’ treatments compared with 87 % during ‘Bat’ treatments, 37 % during ‘LED’ treatments and 43 % during the ‘Control’. The proportion of moths responding was significantly higher on the unlit ‘Bat’ treatment compared with both of the bat-free treatments: ‘Control’ (SE = 0.9727, z = 2.354, p = 0.019) and ‘LED’ (SE = 0.895, z = 2.897, p = 0.004). There was no statistically significant difference between the two bat-free treatments, ‘Control’ vs. ‘LED’ (SE = 0.705, z = 0.431, p = 0.666).

**Supplementary Pictures**


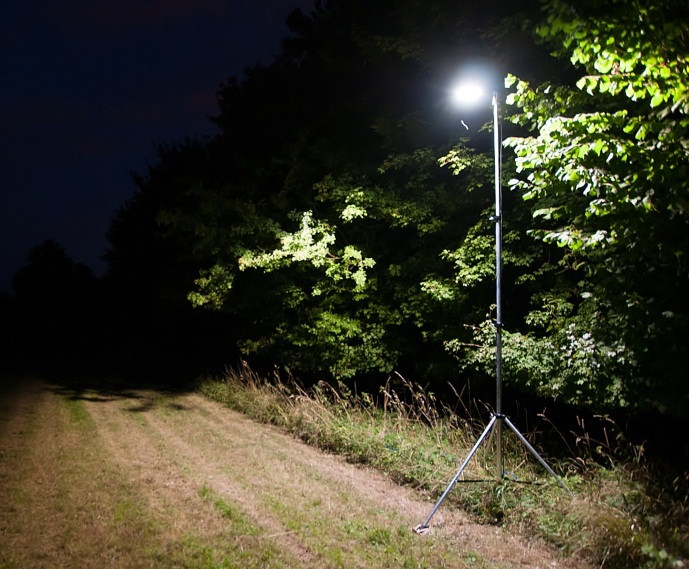


**Figure S1**. Positioning of street light in relation to the woodland edge.

**Figure S2**. Front (left) and back (right) of the ultrasound playback and moth recording equipment.

**Table S1**. Model comparison data.

| Model | Terms | Description of effects | AIC | Δ deviance |
| --- | --- | --- | --- | --- |
| GLMM1 | Powerdives ~ 1 + (1 | Site) | Fixed = None  Random = Site | 101.89 | 97.89 |
| GLMM2 | Powerdives ~ Treatment + (1 | Site) | Fixed = Treatment  Random = Site | 89.14 | 79.14 |
| GLMM3 | Powerdives ~ Treatment | Fixed = Treatment  Random = None | 89.83 | - |

**Table S2**. Percentage of moths responding (either changing flight direction or powerdiving) per treatment.

|  | Percentage of Moths Responding | |
| --- | --- | --- |
| Treatment | Responded | Did not respond |
| Control | 43 | 57 |
| Bat | 87 | 13 |
| LED | 37 | 63 |
| LED-Bat | 58 | 42 |

**References**

1. Holderied, M. W. & von Helversen, O. 2003 Echolocation range and wingbeat period match in aerial-hawking bats. *Proc. R. Soc. Lond. B* **270**, 2293–2299. (doi:10.1098/rspb.2003.2487)

2. Rydell, J., Skals, N., Surlykke, A. & Svensson, M. 1997 Hearing and bat defence in geometrid winter moths. *Proc. R. Soc. Lond. B* **264**, 83–88. (doi:10.1098/rspb.1997.0012)

3. Svensson, A. M. & Rydell, J. 1998 Mercury vapour lamps interfere with the bat defence of tympanate moths (*Operophtera* spp.; Geometridae). *Anim. Behav.* **55**, 223–226. (doi:10.1006/anbe.1997.0590)

4. Briscoe, A. D. & Chittka, L. 2001 The evolution of color vision in insects. *Annu. Rev. Entomol.* **46**, 471–510. (doi:10.1146/annurev.ento.46.1.471)

5. Crawley, M. J. 2008 *The R Book*. Chichester: John Wiley & Sons, Ltd.
